# Shengmai Injection Improved Doxorubicin-Induced Cardiomyopathy by Alleviating Myocardial Endoplasmic Reticulum Stress and Caspase-12 Dependent Apoptosis

**DOI:** 10.1155/2015/952671

**Published:** 2015-03-09

**Authors:** Yu Chen, Yong Tang, Yin Xiang, Yu-Quan Xie, Xiao-Hong Huang, Ya-Chen Zhang

**Affiliations:** Division of Cardiology, Xin Hua Hospital Affiliated to Shanghai Jiao Tong University School of Medicine, 1665 Kongjiang Road, Shanghai 200092, China

## Abstract

*Background.* Apoptosis plays vital roles in the progression of doxorubicin-induced cardiomyopathy (DOX-CM). Endoplasmic reticulum stress (ER stress) could induce specific apoptosis by caspase-12 dependent pathway. Shengmai Injection (SMI), a famous Traditional Chinese Medicine, could alleviate the heart damage via inhibiting myocardial apoptosis. However, it is unknown whether SMI can alleviate ER stress and its specific apoptosis in the setting of DOX-CM. *Objective.* To explore the effects of SMI on heart function, myocardial ER stress, and apoptosis of DOX-CM rats. *Methods.* Rats with DOX-CM were treated by SMI. Heart function was assessed by echocardiography and brain natriuretic peptide. Myocardial apoptosis was detected by TUNEL assay. ER stress was assessed by detecting the expressions of GRP78 and caspase-12. *Results.* At the end of eight-week, compared to control, significant heart dysfunction happened in DOX group. The ratio of apoptotic cardiomyocytes and the expressions of GRP78 and caspase-12 increased significantly (*P* < 0.05). Compared to DOX group, the apoptotic ratio and the expressions of GRP78 and caspase-12 significantly decreased in DOX + SMI group (*P* < 0.05), accompanied with improved heart function. *Conclusion.* SMI could alleviate myocardial ER stress and caspase-12 dependent apoptosis, which subsequently helped to improve the heart function of rats with DOX-CM.

## 1. Introduction

Doxorubicin (DOX) is a commonly used chemotherapeutic in clinic. However, its application was greatly limited by the cardiotoxicity, which could lead to doxorubicin-induced cardiomyopathy (DOX-CM), one of the severest complications of DOX [1, 2]. With dose-dependent and irreversible myocardial damage and heart function degeneration, the patients with DOX-CM have a 1-year survival rate of less than 50 percent [3].

The pathogenesis of DOX-CM has not been fully clarified yet. Multiple factors are involved in the mechanisms of DOX-CM, such as free radical damage and calcium overload [1, 2]. Myocardial apoptosis plays a vital role in the progression of DOX-CM [4], whereby attenuating myocardial apoptosis could improve left ventricular function [5].

As a common pathway of many other stresses, endoplasmic reticulum stress (ER stress) is widely involved in the development of cardiovascular system diseases [6, 7]. External and internal stimuli, such as hypoxia, toxicant, and oxidative stress, can activate ER stress. Moderate ER stress plays a positive role in maintaining ER function and homeostasis by enhancing protein folding capacity with increased expression of ER chaperones glucose-regulated protein 78 (GRP78) and GRP94. Excessive ER stress can cause cell injury, death, and apoptosis. Recent studies found that ER stress existed in heart failure and contributed to the myocardial apoptosis [8, 9]. However, the roles of ER stress in apoptosis in DOX-CM have not been reported.

Shengmai injection (SMI), a famous traditional Chinese medicine (TCM), has long been used to treat heart failure in China [10, 11]. Studies demonstrated that SMI could alleviate the myocardium injury and heart dysfunction of patients treated with DOX [12, 13]. In rats with DOX-CM SMI has been proven to exert a cardioprotective effect by inhibiting cardiomyocyte apoptosis [14, 15]. However, whether SMI could alleviate myocardial ER stress and ER stress specific apoptosis remains unknown. In this study, we explored the effects of SMI on heart function, myocardial ER stress, and apoptosis of DOX-CM rats.

## 2. Methods and Materials

### 2.1. Ethics Statement

All experimental procedures were approved by the Institutional Authority for Laboratory Animal Care of Xin Hua Hospital Affiliated to Shanghai Jiao Tong University School of Medicine and conformed to the Guide for the Care and Use of Laboratory Animals published by the National Institutes of Health (NIH Publication, Eighth Edition, 2011).

### 2.2. Animal

Sixty male Sprague-Dawley rats (230 ± 10 g, eight weeks) were purchased from SLAC Laboratories (Shanghai, China). All rats were housed with appropriate humidity (50–60%) and temperature (20–25°C), exposed to a 12-hour light and dark cycle, and fed with standard chow and tap water ad libitum. The cages were kept dry and clean. The rats were randomized into control group (*n* = 20), DOX group (*n* = 20), and DOX + SMI group (*n* = 20). In DOX group, the rats were injected intraperitoneally (i.p.) with DOX (Sigma, Saint Louis, USA) in six equal injections (each containing 2.5 mg/kg DOX) within two weeks according to previous study [16] and then followed for six weeks. In DOX + SMI group, DOX of the above doses was injected i.p. within two weeks. SMI (Hehuang, Shanghai, China) was injected i.p. in 12 equal injections (each containing 3 mL/kg SMI according to clinical dosage) within four weeks. In first two weeks, DOX and SMI were alternately injected i.p., and then SMI alone was administrated. Subsequently the rats of DOX + SMI group were followed for four weeks. Control group was administered i.p. with isometric saline in the first four weeks and then followed for another four weeks. In other words, all rats in the three groups were followed for eight weeks since they received the first injection.

### 2.3. Serum BNP Assay

Brain natriuretic peptide (BNP) is a biomarker of congestive heart failure, which has been widely used to assess the severity of cardiac dysfunction in clinic. Rats were anesthetized with chloral hydrate (300 mg/kg, i.p.) at the end of the follow-up duration. Blood samples were collected via the tail vein and centrifuged to obtain serum. Then the BNP concentration was detected by BNP EIA Kit in accordance with the manufacturer's instructions (Sigma, Saint Louis, USA).

### 2.4. Echocardiography

After blood sample collection, transthoracic echocardiography was performed by Vevo 770 Micro-Ultrasound System (VisualSonics, Toronto, Canada) with a cardiac probe (RMV716). After the anterior and left lateral thoracic regions were shaved, rats were placed in the supine position. Ultrasound transonic gel was placed on the thorax to optimize visibility. M-mode and two-dimensional echocardiographic images were recorded at the level of the papillary muscles. Left ventricular (LV) anterior wall thickness (LVAW), LV posterior wall thickness (LVPW), and LV internal dimension (LVID) were measured in systole and diastole. Ejection fraction (EF), fractional shortening (FS), and LV volume at end-systole (LVESV) and end-diastole (LVEDV) were calculated from the above parameters.

### 2.5. Myocardium Sample Collection

After echocardiography, rats were sacrificed by intravenous injection of potassium chloride (2 mmol/Kg). Hearts were rapidly harvested and sliced into two parts. The apical part was frozen with liquid nitrogen and kept at −80°C until real-time fluorescence quantitative PCR and Western blotting analyses. The other part was fixed with formalin for TUNEL assay.

### 2.6. Real-Time Quantitative PCR for GRP78 and Caspase-12 mRNA

The total RNA of myocardial cells was extracted with TRIzol Reagent (Invitrogen, Carlsbad, CA, USA). cDNA was synthesized using a Takara RNA PCR Kit (Takara, Dalian, China). The following oligonucleotides were used as primers. GRP78: 5′-CCA­CCA­GGA­TGC­AGA­CAT­TG-3′ (forward), 5′-AGG­GCC­TCC­ACT­TCC­ATA­GA-3′ (reverse). Caspase-12: 5′-CAC­TGC­TGA­TAC­AGA­TGA­GG-3′ (forward), 5′-CCA­CTC­TTG­CCT­ACC­TTC­C-3′ (reverse) (synthesized by Invitrogen, Carlsbad, CA, USA). Thermal cycling conditions were 95°C, 15 s, 56°C, 15 s, 72°C, 45 s, and 40 cycles. *β*-actin was utilized as internal control.

### 2.7. Western Blotting

Protein was extracted from cardiac tissue homogenates with ice-cold RIPA buffer and protease inhibitors (Roche, Basel, Switzerland). Protein concentration was determined by the Bradford protein assay (Beyotime, Nantong, China). Equal amounts (30–50 *μ*g) of proteins were separated by a 10% sodium dodecyl sulfate-polyacrylamide gel electrophoresis (SDS-PAGE) and transferred to a polyvinylidene fluoride (PVDF) membrane (Millipore, Billerica, MA, USA). After being blocked with 5% skim milk in Triethanolamine-Buffered Saline Solution with Tween (TBST), the membranes were incubated with 1 : 1000 dilution of primary antibodies against GRP78 and caspase-12 (Cell Signaling Technology, Danvers, Massachusetts, USA) at 4°C overnight. After three washes in TBST, the membranes were incubated with horseradish peroxidase- (HRP-) conjugated secondary antibodies (Cell Signaling Technology, Danvers, Massachusetts, USA) at a 1 : 1000 dilution at room temperature for 1 hour. After three washes in TBST, the protein bands were detected by enhanced chemiluminescence (ECL) reagents (Millipore, Billerica, MA) and quantified with the ChemiDoc XRS imaging system (Bio-Rad, Hercules, CA, USA).

### 2.8. TUNEL Assay

TUNEL assay was utilized to detect the apoptosis in cardiac tissues. Fresh myocardial tissues of rats were fixed with formalin, dehydrated, transparent, embedded in paraffin, and cut into 5–8 *μ*m thick slices. Then the myocardial sections were deparaffinized, rehydrated, and pretreated with 20 *μ*g/mL proteinase K. The endogenous peroxidase was blocked by 3% hydrogen peroxide. The sections were subsequently incubated with terminal deoxynucleotidyl transferase (TdT) reaction mix at 37°C for 1 hour, followed by antidigoxigenin antibody at 37°C for 30 min. After washing, slides were incubated with streptavidin-biotin-peroxidase for 20 min, stained with 3,3′-diaminobenzidine tetrahydrochloride, and counterstained with hematoxylin. Finally, the sections were dehydrated, coverslipped, and observed. Eight sights of each section were randomly selected under microscope. The number of TUNEL-positive cardiomyocyte nuclei and the total cardiomyocyte nuclei in each sight were counted. The ratio of apoptotic cardiomyocytes was calculated by dividing the number of TUNEL-positive cardiomyocyte nuclei by the number of total cardiomyocyte nuclei.

### 2.9. Statistical Analysis

Data were expressed as mean ± SD or frequency. Differences between any two groups were analyzed by independent *t*-tests with SPSS 17.0 software (SPSS Inc., Chicago, USA). A *P* ≤ 0.05 was considered as significant difference.

## 3. Results

### 3.1. General Appearance

**Figure 1:**
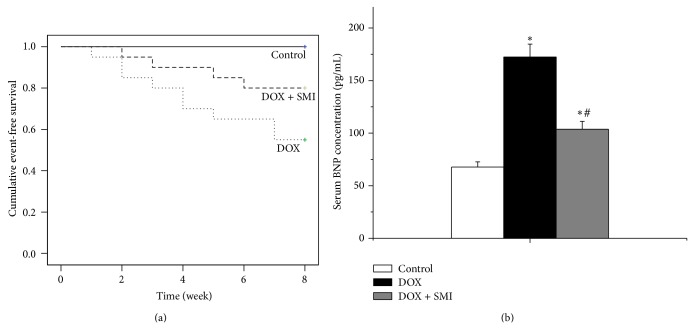
Survival and serum BNP concentration across all groups: (a) survival and (b) serum BNP concentration. At the end of follow-up duration, there were 20 survivals in control group, 11 in DOX group, and 16 in DOX + SMI group, respectively. Compared to control group, ^*^ *P* < 0.05; compared to DOX group, ^#^ *P* < 0.05.

At the end of eight weeks, all the rats survived in control group. 11 rats (55.0%) survived in DOX group, whereas 16 ones survived (80%) in DOX + SMI group (Figure 1(a)). Ascites, a symptom of heart failure, occurred in seven rats in DOX group, whereas only three rats in DOX + SMI group experienced ascites.

### 3.2. Serum BNP Concentrations

As a biomarker of congestive heart failure, the serum BNP concentration of DOX group was significantly higher than those of control group and DOX + SMI group at the end of follow-up duration (*P* < 0.05), Figure 1(b). The serum BNP concentration of DOX + SMI group was higher than that of control group, but the difference was not significant in statistics (*P* > 0.05).

### 3.3. Echocardiography

**Figure 2:**
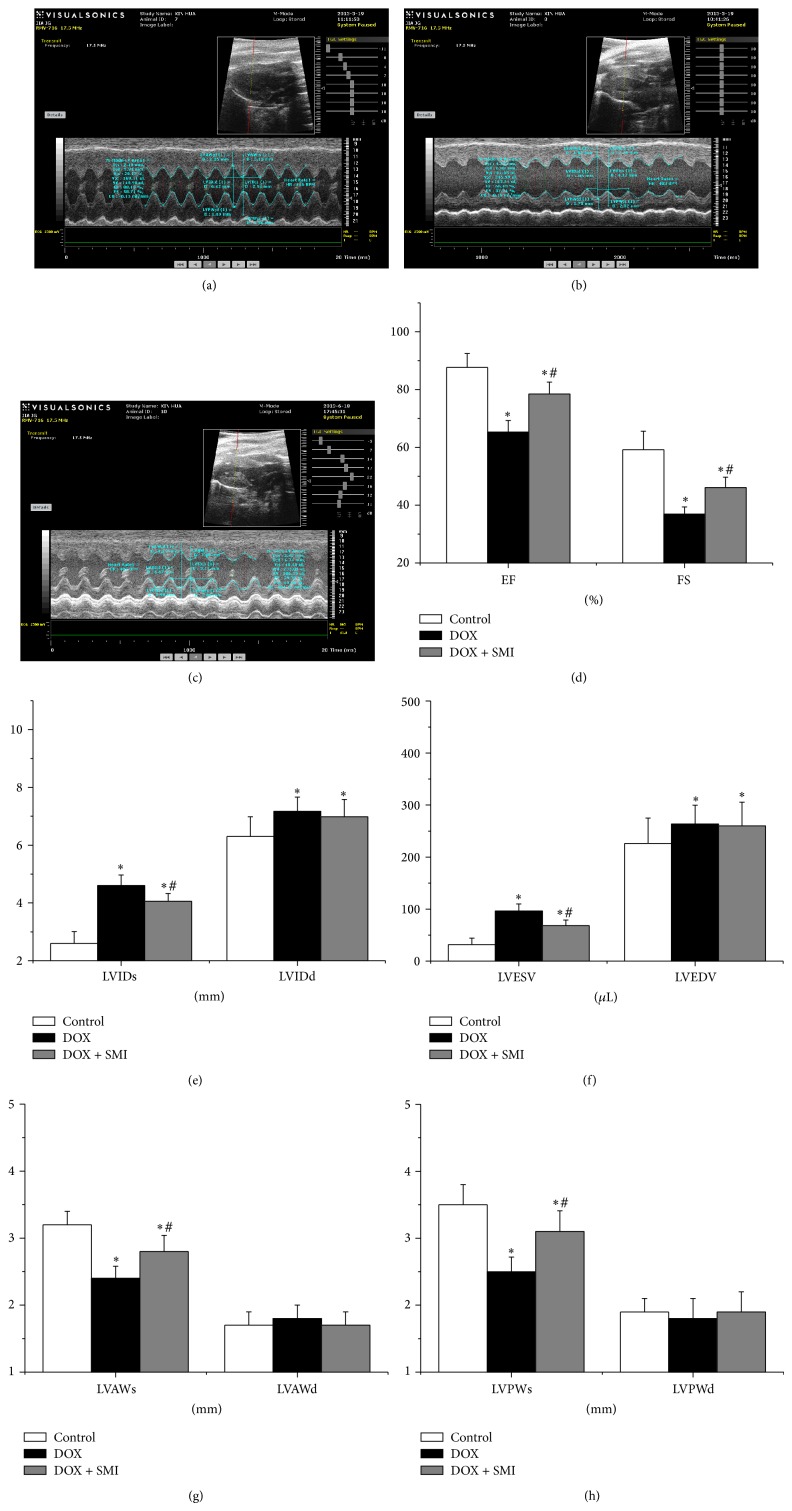
Echocardiography across all groups: (a) control group, (b) DOX group, (c) DOX + SMI group, (d) EF and FS, (e) LVIDs and LVIDd, (f) LVESV and LVEDV, (g) LVAWs and LVAWd, and (h) LVPWs and LVPWd. At the end of follow-up duration, there were 20 survivals in control group, 11 in DOX group, and 16 in DOX + SMI group, respectively. Compared to control group, ^*^ *P* < 0.05; compared to DOX group, ^#^ *P* < 0.05.

The heart changes of rats detected by echocardiography were shown in Figure 2. LVIDs, LVIDd, LVESV, and LVEDV in DOX group were significantly greater than those of control group (*P* < 0.05), while LVAWs, LVPWs, EF, and FS were significantly less than those of control group (*P* < 0.05). Moreover, LVIDs, LVAWs, LVPWs, LVESV, EF, and FS in DOX + SMI group were improved significantly compared to those of DOX group (*P* < 0.05).

### 3.4. Myocardial Apoptosis Detected by TUNEL Assay

**Figure 3:**
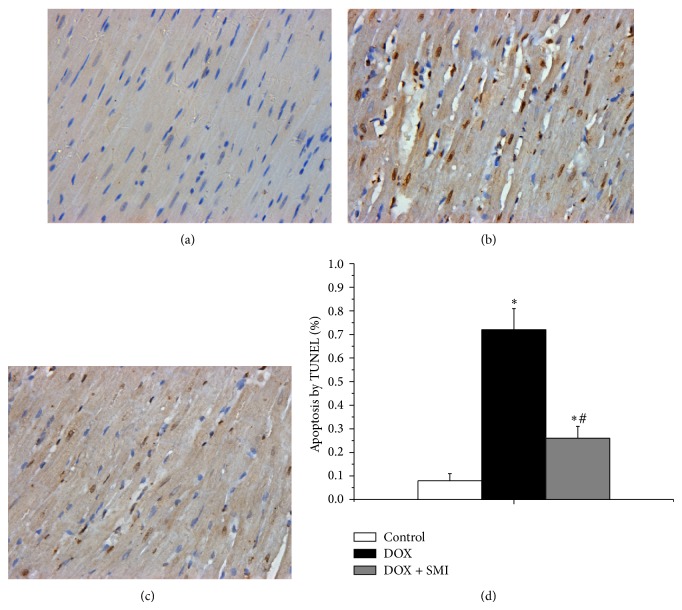
TUNEL assay across all groups (×400): (a) control group, (b) DOX group, (c) DOX + SMI group, and (d) quantitative analysis of myocardial apoptosis across all groups. The apoptotic nuclei were stained brown, while the normal ones were blue. At the end of follow-up duration, there were 20 survivals in control group, 11 in DOX group, and 16 in DOX + SMI group, respectively. Compared to control group, ^*^ *P* < 0.05; compared to DOX group, ^#^ *P* < 0.05.

The TUNEL assay showed that DOX induced apoptosis in the myocardium of rats. As shown in Figure 3, the ratio of apoptotic cardiomyocytes in DOX group was 0.72 ± 0.09%, which was significantly higher than that that in control group (0.08  ±  0.03%, *P* < 0.05). In DOX + SMI group, the apoptotic ratio was 0.26 ± 0.05%, which was significantly lower than that in DOX group (*P* < 0.05). Besides, the apoptotic ratio in DOX + SMI group was higher than that in control group (*P* < 0.05).

### 3.5. Expression of GRP78 mRNA Detected by Real-Time Fluorescence Quantitative PCR

**Figure 4:**
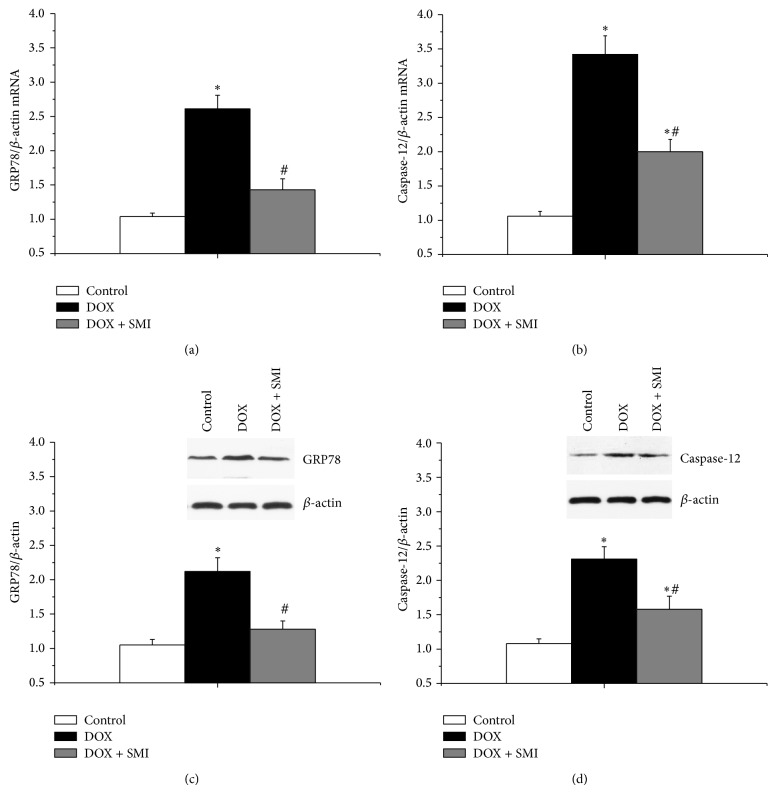
The expressions of GRP78 and caspase-12 cross all the groups: (a) GRP78 mRNA expression, (b) caspase-12 mRNA expression, (c) GRP78 protein expression, and (d) caspase-12 protein expression. At the end of follow-up duration, there were 20 survivals in control group, 11 in DOX group, and 16 in DOX + SMI group, respectively. Compared to control group, ^*^ *P* < 0.05; compared to DOX group, ^#^ *P* < 0.05.

The myocardial expressions of GRP78 mRNA across all groups were shown in Figure 4(a). The myocardial expression of GRP78 mRNA of DOX group was significantly greater than that of control group (*P* < 0.05). The myocardial expression of GRP78 mRNA of DOX + SMI group was significantly lower than that of DOX group (*P* < 0.05). Compared to that of control group, the myocardial expression of GRP78 mRNA of DOX + SMI group increased, but the difference was not significant in statistics (*P* > 0.05).

### 3.6. Expression of Caspase-12 mRNA Detected by Real-Time Fluorescence Quantitative PCR

The myocardial expressions of caspase-12 mRNA across all groups were shown in Figure 4(b). Both of the myocardial expressions of caspase-12 mRNA in rats of DOX group and DOX + SMI group were significantly greater than that of control group (*P* < 0.05). Furthermore, the myocardial expression of caspase-12 mRNA in rats of DOX + SMI group was significantly lower than that of DOX group (*P* < 0.05).

### 3.7. GRP78 Expression Detected by Western Blotting

The myocardial expressions of GRP78 protein across all groups were shown in Figure 4(c). The myocardial expression of GRP78 protein of DOX group was significantly higher than that of control group (*P* < 0.05). Besides, the myocardial expression of GRP78 protein of DOX + SMI group was lower than that of DOX group (*P* < 0.05). There was no significant difference between DOX + SMI group and control group (*P* > 0.05).

### 3.8. Caspase-12 Expression Detected by Western Blotting

The myocardial expressions of caspase-12 protein across all groups were shown in Figure 4(d). Both of the myocardial expressions of caspase-12 protein of DOX group and DOX  + SMI group were significantly greater than that of control group (*P* < 0.05). Moreover, the myocardial expression of caspase-12 protein of DOX + SMI group was lower than that of DOX group (*P* < 0.05).

## 4. Discussion

In this study, low survival and significant heart failure happened in rats with DOX-CM. The damaged heart structure and function were proven by deteriorating echocardiographic features and increased BNP level. Furthermore, significant myocardial apoptosis and ER stress occurred in rats with DOX-CM which were evidenced by TUNEL assay and increased expressions of GRP78 and caspase-12. Moreover, SMI improved the heart function and survival of rats with DOX-CM and alleviated the myocardial ER stress and apoptosis caused by DOX.

Dilated cardiomyopathy (DCM) is the main feature of DOX-CM, characterized by declined heart function, dilated ventricular chamber, and thinner wall [1, 2]. Our results showed that EF and FS reduced significantly in DOX group, indicating the declined heart systolic function. LVID, LVESV, and LVEDV increased in DOX group, indicating the dilated heart ventricle. LVAWs and LVPWs reduced in DOX group, showing the thinner ventricular wall. All of the above complied with the characteristics of DCM. Most of these parameters were reversed or alleviated by SMI, implying that SMI can improve the heart function and structure of rats with DOX-CM.

Cardiomyocyte apoptosis plays an important role in the progression of DOX-CM. Cardiomyocyte apoptosis causes the proliferation of neighboring fibrous tissue and compensatory hypertrophy of neighboring cardiomyocytes, which lead to cardiac remodeling and heart insufficiency [17, 18]. Kumar et al. found that the rats with DOX-CM experienced significant myocardial apoptosis, which was evidenced by increased expressions of Bax and caspase-3 [4]. In our study DOX group showed significant cardiomyocyte apoptosis proven by TUNEL assay, which was accordant with Kumar et al.'s study. Furthermore, DOX + SMI group showed less apoptotic cells than DOX group, implying SMI could alleviate myocardium apoptosis.

GRP78, an indicator of ER stress, increased significantly in DOX group, showing the activation of ER stress in the heart of rats with DOX-CM. ER stress can induce apoptosis via c-Jun N-terminal kinase (JNK), C/EBP homologous protein (CHOP), and caspase-12 dependent pathways [19]. As a specific proapoptotic pathway, ER stress could activate caspase-12, spur a cascade of caspase, and thereby induce apoptosis [20]. Lou et al. found caspase-12 induced myocardial apoptosis in rats with myocardial infarction [21]. In our study, the increased GRP78 and caspase-12 in DOX group implied that ER stress induced myocardial apoptosis by activating caspase-12 dependent pathway. Furthermore, both of GRP78 and caspase-12 declined significantly in DOX + SMI group compared to DOX group, which suggested that SMI suppresses ER stress and caspase-12 dependent apoptosis.

SMI is based on a famous Chinese prescription “Shengmai San” (SMS), which consists of* ginseng*,* radix ophiopogonis,* and* schisandra chinensis* and is officially recorded in Chinese Pharmacopoeia (version 2010). In China SMI has been used to treat heart disease including heart failure, myocardium infarction, and hypotension [11, 22, 23]. Clinical studies found that SMI could improve the heart function and exercise capacity of patients with myocardial infarction, DCM, and DOX-CM [10, 13, 16]. Experiments in vivo and vitro demonstrated that SMI and SMS could alleviate oxidative stress and myocardial cell apoptosis [15, 24, 25]. To our knowledge, our study first found that SMI could inhibit myocardial ER stress and caspase-12 dependent apoptosis, which shed light on a new pharmacological mechanism of SMI for treating DOX-CM.

## 5. Conclusion

There were significant myocardial ER stress and apoptosis in rats with DOX-CM, as well as declined heart function and survival. SMI could alleviate myocardial ER stress and ER stress specific apoptosis by inhibiting caspase-12 dependent pathway, which subsequently helped to improve cardiac function of rats with DOX-CM. These findings provide new insights into the DOX-CM pathogenesis and suggest SMI as a potential agent to treat the cardiac damage of DOX.

## References

[1] Octavia Y., Tocchetti C. G., Gabrielson K. L., Janssens S., Crijns H. J., Moens A. L. (2012). Doxorubicin-induced cardiomyopathy: from molecular mechanisms to therapeutic strategies. *Journal of Molecular and Cellular Cardiology*.

[2] Chatterjee K., Zhang J., Honbo N., Karliner J. S. (2010). Doxorubicin cardiomyopathy. *Cardiology*.

[3] Takemura G., Fujiwara H. (2007). Doxorubicin-induced cardiomyopathy: from the cardiotoxic mechanisms to management. *Progress in Cardiovascular Diseases*.

[4] Kumar D., Kirshenbaum L. A., Li T., Danelisen I., Singal P. K. (2001). Apoptosis in adriamycin cardiomyopathy and its modulation by probucol. *Antioxidants & Redox Signaling*.

[5] Xu W. H., Son J., Wang Y. (2006). Granulocyte colony-stimulating factor reduces cardiomyocyte apoptosis and improves cardiac function in adriamycin-induced cardiomyopathy in rats. *Cardiovascular Drugs and Therapy*.

[6] Minamino T., Kitakaze M. (2010). ER stress in cardiovascular disease. *Journal of Molecular and Cellular Cardiology*.

[7] Groenendyk J., Sreenivasaiah P. K., Kim D. H., Agellon L. B., Michalak M. (2010). Biology of endoplasmic reticulum stress in the heart. *Circulation Research*.

[8] Jang Y. M., Kendaiah S., Drew B. (2004). Doxorubicin treatment in vivo activates caspase-12 mediated cardiac apoptosis in both male and female rats. *FEBS Letters*.

[9] Szegezdi E., Duffy A., O'Mahoney M. E. (2006). ER stress contributes to ischemia-induced cardiomyocyte apoptosis. *Biochemical and Biophysical Research Communications*.

[10] Zhang Y.-C., Chen R.-M., Zhao M.-H. (2002). Effect of shengmai injection on hemodynamics in patients with dilated cardiomyopathy. *Zhongguo Zhong Xi Yi Jie He Za Zhi*.

[11] Chen J., Yao Y., Chen H., Kwong J. S. W. (2012). Shengmai (a traditional Chinese herbal medicine) for heart failure. *Cochrane Database of Systematic Reviews*.

[12] Yang X. L. (2008). Clinical observation on the prevention of doxorubicin-associated cardiotoxicity by Shengmai injection. *Guide of China Medicine*.

[13] Zhang Y. K., He W. B., Zhang H., He H. Y., Xie J., Xu J. P. The observation of prevention effect of Shengmai injection for acute adriamycin induced cardiotoxicity. *Journal of Emergency in Traditional Chinese Medicine*.

[14] Jin H., Sun L. M. (2006). Protective effects of Shengmai injection on myocardium injury induced by adriamycin in rats. *Lishizhen Medicine and Materia Medica Research*.

[15] Yang X. D., Shu W. Q., Yang S. B. (2008). Protective effect of Shengmai injection on adriamycin-induced myocardium injury in rats and its mechanism. *Central South Pharmacy*.

[16] Zhang Y.-C., Chen R.-M., Lu B.-J., Rong Y.-Z. (2008). Effect of Shengmai Injection on cardiac function and inflammatory reaction in patients with acute coronary syndrome. *Chinese Journal of Integrative Medicine*.

[17] French B. A., Kramer C. M. (2007). Mechanisms of postinfarct left ventricular remodeling. *Drug Discovery Today: Disease Mechanisms*.

[18] Sabbah H. N. (2000). Apoptotic cell death in heart failure. *Cardiovascular Research*.

[19] Xu C., Bailly-Maitre B., Reed J. C. (2005). Endoplasmic reticulum stress: cell life and death decisions. *The Journal of Clinical Investigation*.

[20] Morishima N., Nakanishi K., Takenouchi H., Shibata T., Yasuhiko Y. (2002). An endoplasmic reticulum stress-specific caspase cascade in apoptosis. Cytochrome *c*-independent activation of caspase-9 by caspase-12. *The Journal of Biological Chemistry*.

[21] Lou L.-X., Wu A.-M., Zhang D.-M. (2014). Yiqi huoxue recipe improves heart function through inhibiting apoptosis related to endoplasmic reticulum stress in myocardial infarction model of rats. *Evidence-based Complementary and Alternative Medicine*.

[22] Chen C.-Y., Lu L.-Y., Chen P. (2013). Shengmai injection, a traditional chinese patent medicine, for intradialytic hypotension: a systematic review and meta-analysis. *Evidence-Based Complementary and Alternative Medicine*.

[23] Gao Z.-Y., Guo C.-Y., Shi D.-Z. (2008). Effect of shengmai injection on the fatality rate of patients with acute myocardial infarction: a systematic review. *Zhongguo Zhong Xi Yi Jie He Za Zhi*.

[24] Ichikawa H., Wang L., Konishi T. (2006). Prevention of cerebral oxidative injury by post-ischemic intravenous administration of Shengmai San. *The American Journal of Chinese Medicine*.

[25] Nishida H., Ichikawa H., Konishi T. (2007). *Shengmai-san* enhances antioxidant potential in C2C12 myoblasts through the induction of intracellular glutathione peroxidase. *Journal of Pharmacological Sciences*.

